# Adjusting for covariates representing potential confounders, mediators, or competing predictors in the presence of measurement error: Dispelling a potential misapprehension and insights for optimal study design with nutritional epidemiology examples

**DOI:** 10.12688/f1000research.152466.1

**Published:** 2024-07-24

**Authors:** Roger S. Zoh, Diana M. Thomas, Carmen D. Tekwe, Xiaoxin Yu, Colby J. Vorland, Nikhil V. Dhurandhar, David M. Klurfeld, David B. Allison

**Affiliations:** 1 Department of Epidemiology and Biostatistics, Indiana University Bloomington, Bloomington, Indiana, 47408, USA; 2 Department of Mathematical Sciences, West Point United States Military Academy, New York, NY, USA; 3 Department of Applied Health Science, Indiana University Bloomington, Bloomington, Indiana, 47408, USA; 4 Department of Nutritional Sciences, Texas Tech University, Lubbock, Texas, USA

**Keywords:** Measurement Error, Bias, Measurement reliability, Type I Error, Covariate Adjustment

## Abstract

**Background:**

Variables such as dietary intake are measured with error yet frequently used in observational epidemiology. Although this limitation is sometimes noted, these variables are still often modeled as covariates without formal correction or sincere dialogue about measurement unreliability potentially weakening the validity of statistical conclusions. Further, larger sample sizes increase power (bias) to detect spurious correlations. Counterintuitively, recent work suggested a *non-monotonic* relationship between confounder unreliability and how much controlling for the confounder reduces (or induces) bias when testing for an exposure-outcome association. If true, such non-monotonicity would be especially concerning for applications such as nutrition, where measurement reliability varies substantially, and large sample sizes are common.

**Methods:**

We offer a detailed derivations of the square partial correlation between the outcome and exposure, controlling for the confounder. In our derivation, the measurement reliabilities of exposures and confounders are not arbitrarily constrained to be equal. Further, our theoretical results are investigated using simulations.

**Results:**

Reassuringly, these derivations and simulations show that the counterintuitive non-monotonicity relationship between confounder unreliability and how much controlling for the confounder reduces (or induces) bias when testing for an exposure-outcome association is an artifact of the arbitrary constraint which forces the measurement reliabilities of exposures and confounders to be equal, which that does not always hold.

**Conclusions:**

The profound and manifold effects of measurement error on estimation and statistical conclusion validity in realistic scenarios indicate that merely mentioning measurement error as a limitation and then dispensing with it is not an adequate response. We also explore questions for optimal study design subject to resource constraints when considering reliability of exposures, covariates, and outcomes.

## Introduction

The challenges of measuring dietary intake and other variables in observational epidemiology in nutrition and obesity research have been widely discussed. ^1^^–^^3^ Data on nutrient or energy intake inform prevention and treatment guidance and policies for many health conditions. Fidelity of data to true nutrient and energy intake is essentially related to the ability to draw valid scientific conclusions about nutritional effects. It is not easy to obtain food intake information, however, particularly from a large number of individuals as is done for the highly reputable and influential National Health and Nutrition Examination Survey (NHANES). NHANES obtains nutrient and energy intake data through self-reported dietary recall questionnaires. This approach involves individuals reporting food and drinks consumed in the past 24 hours followed by detailed questions to accurately gauge the amounts of nutrients and energy consumed. Another such subjective approach that is popular is the food-frequency questionnaire, which asks individuals about the frequency of consumption of various foods.

Understandably, such information related to nutrient and energy intake is influenced by intentional or unintentional misreporting, which substantially impacts accuracy. For example, a study pooled data from five large studies that had examined the validity of food-frequency questionnaires and 24-h dietary recalls against recovery biomarkers. Across this diverse sample of Americans, subjective estimates of energy intake explained less than 10% of the variance in true energy intake. ^1^ It appears that the self-reported measures systematically underestimate energy intake by hundreds of kcal per day, and the NHANES surveys may underreport energy intake by as much as 800 kcal per day. ^2^^,^^3^

Accuracy of data should be a concern for any researcher, and this level of inaccuracy should be disconcerting. However, it is a common practice to acknowledge the limitations of such measurements and to then carry on with the research as though it has at least some validity despite these limitations. Some common practices to dismiss or minimize concerns around lack of accuracy of data include rationalization that self-reported data about energy or nutrient intake have good reproducibility, or that they could be adjusted by a common factor. ^4^ There are serious counterarguments against such rationalizations. Settling for reproducibility of data instead of accuracy is like using a mismarked measuring tape. One can reproduce the result, but it is still inaccurate. Also, dietary underreporting varies with the type of food consumed, gender, age, smoking habits, social class, education level, dietary restraints, body mass index (BMI) of the respondents, and other life stage factors, ^5^ which makes it implausible that one could adequately correct a given dataset by a single factor and arithmetic operation.

Considering the serious implications of using decidedly inaccurate information, some investigators have raised concerns that some limitations are so severe that we are better off not conducting certain research or not using certain methods at all until more accurate methods become available. For example, Lear wrote “One may wonder if we should stop nutritional research altogether until we can get it right”. ^4^ While this may be an extreme rhetorical position, Dhurandhar et al. ^5^ noted that in some cases, measurement properties are so bad that it is better to not perform the research at all than to use the poor measurement instruments (even if the instruments are the best available for a particular context). Putting it succinctly, on some occasions, *something is not better than nothing.*

Recently, we became aware of a particular finding regarding the effects of controlling for a confounding variable that is measured with error. Westfall and Yarkoni ^6^ showed results that seemed to imply that there is not necessarily a monotonic relationship between **(a)** the degree of measurement error in a confounding variable and **(b)** the extent to which controlling for the measurement of the confounding variable reduces bias in testing for an exposure association with an outcome. Rather, the degree of bias in testing appears to increase as one moves from a completely unreliable measure of the confounder to a partially reliable measure and then decreases again as one approaches a perfectly reliable measure.

Note that in frequentist significance testing (the type of testing generally used in this journal), a test may be said to be biased when the sampling distribution of *P* values from applying the test is not uniform on the interval [0,1] under the null hypothesis. ^7^ The main concern is that the “size of the test” is inflated such that, in probability, too many small *P* values are generated, leading to too many type I errors.

Another key point from the article by Westfall and Yarkoni ^6^ that appears counterintuitive is that the larger the sample size, the higher the error rate. This is critical in nutritional epidemiology because greater importance is regularly given to the largest cohort studies when in fact the largest studies may be the most susceptible to biasing in significance testing both because they have more power to detect all effects, including spurious ones, and because large scale studies may be less well able to measure variables well. ^8^

Although we were able to identify some articles in the nutrition and obesity literature that cited the Westfall and Yarkoni article, ^9^^–^^17^ none of them addressed the issue of modest reliability potentially being worse than poorer reliability. To the best of our ability to discern, this issue has not been recognized in the nutrition and obesity literature. Yet, this methodologic issue in which intermediary measurement reliability may be worse than either very low or very high reliability seems especially concerning for the nutritional epidemiology field, where even aspects of dietary intake that can be measured with some degree of reliability and validity often have very modest degrees of reliability or validity. ^18^ Thus, this non-monotonicity problem may be a particular concern, and if true, might suggest the startling conclusion that in nutrition epidemiology it is better to not measure some covariates at all than to include them in the study, if they have only modest reliability.

Here we provide an explanation of the phenomenon in simple scenarios so that researchers, reviewers, and editors in the nutrition and obesity research communities can see that the conclusion about non-monotonicity of bias as a function of covariate reliability holds only under specific and arguably contrived circumstances. Awareness of this offers some modest reassurance and also interesting insights into the design, analysis, and interpretation of studies.

## Derivation

Consider the following extension to simple linear classical measurement error model

$$\begin{array}{c} Y=\mathrm{\beta Z}+\epsilon_{y}\\ X=\mathrm{\gamma Z}+\epsilon_{x}\\ \begin{array}{c} Z^{\prime }=\mathrm{\alpha Z}+e\\ Z∼N\left(0,1\right) \end{array} \end{array}$$ Model 1

where we assume that the error terms $\epsilon_{y},\epsilon_{x},\text{and}\;e\;$ are all independent of each other and of Z and have a normal distribution with mean zero and finite variance. Without loss of generality, we choose the error variances so that Y , X , and $Z^{\prime }$ each have standard deviation 1. We assume that Y , X , and $Z^{\prime }\;$ are observed, but Z is unobserved. We note that Model 1 is a more general model than the classical measurement error model. Namely, Model 1 agrees with a classical measurement error model only when α=1 .

**Figure 1.**
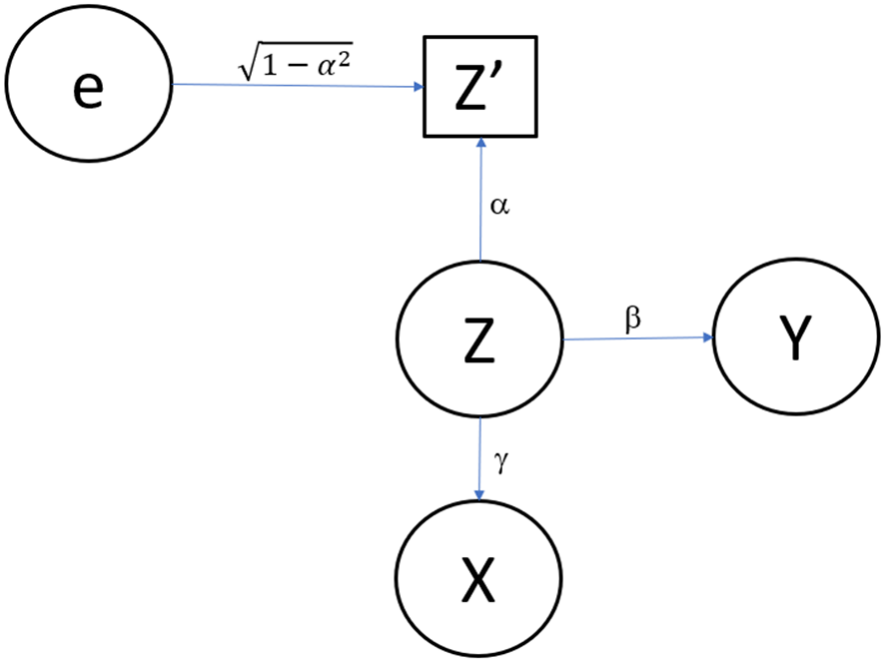
Path diagram for Model 1. Simple model including one confounder measured with error.

Additionally, we depict Model 1 in Figure 1 (omitting the error terms for Y , X , and $Z^{\prime }$ ).

In Figure 1, X represents the independent variable (i.e., exposure, predictor, the putatively causal factor of interest), which could be a measured nutrient, a treatment received or not received, a personal factor, or any other measurable quantity. Z represents a confounding variable which we assume is not observed. Again, Z could be any measurable variable, particularly an aspect of dietary intake. Random measurement error is represented by e . Here we consider classic true score phenomenon, which is a simple conceptualization of measurement error. We denote the error-contaminated measure of Z as $Z^{\prime }$ , with $Z^{\prime }\;$ being a simple additive function of Z and e , where e is independent of Z .

“Classical test theory, also known as true score theory, assumes that each person has a true score, T, that would be obtained if there were no errors in measurement. A person’s true score is defined as the expected score over an infinite number of independent administrations of the scale. Scale users never observe a person’s true score, only an observed score …. It is assumed that observed score … = true score (T) plus some error (E) …. It is also assumed that … the expected value of such random fluctuations (i.e., mean of the distribution of errors over a hypothetical infinite number of administrations on the same subject) is taken to be 0. In addition, random errors are assumed to be uncorrelated with a true score, with no systematic relationship between a person’s true score and whether that person has positive or negative errors”. ^19^

Finally, Y represents the outcome variable (i.e., the dependent variable, the thing to be predicted).

## Confounding, direct effects in mediation, and incremental predictive validity

It is noteworthy that:

(a) With minor modifications, the model depicted in Figure 1 can specify a situation of confounding, causal mediation, or correlated predictors.
(b) The resulting correlation matrix among the variables depicted will be unchanged as a result of these modifications.
(c) The same statistical test will be needed when testing for an association while controlling for confounding, testing for direct effects beyond mediated effects in a mediation model, or testing for incremental predictive validity. ^6^^,^^20^^,^^21^

For example, in the model as depicted, *Z* is a confounder of the association between *X* and *Y*, and the test of interest is whether the squared partial correlation of *X* and *Y* after controlling for *Z* is not zero. However, if we reverse the direction of the arrow from *Z* to *X* (the arrow labeled with the coefficient γ), we are describing a mediation model ^22^ in which *Z* is the hypothesized mediator and the test for the *direct effect* of *X* on *Y* can again be whether the squared partial correlation of *X* and *Y* after controlling for *Z* is not zero. ^23^ As an example, Feng et al. ^24^ ^,p. 3336^ assessed both the direct effects and “the potential indirect effect of sodium intake on blood pressure via body mass index.” That is, Feng et al. modeled BMI as the mediator of sodium intake on systolic and diastolic blood pressure and performed mediation analysis to evaluate the total effect, direct effect, and indirect effect via BMI.

Finally, if we replace the arrow from *Z* to *X* with a double-headed arc, ^25^ we are not specifying the causal relation between *X* and *Z*, only that they are correlated. In this case, we can then test whether, after accounting for the predictive ability of *Z*, *X* can add to the ability to predict *Y.* For example, Pichler et al. ^26^ ^,p. 616^ aimed “to assess the accuracy and precision of a BIA [bioimpedance analysis] device and the relative contribution of BIA *beyond the anthropometric parameters* [emphasis added].”

Given points a, b, and c above, the implications of the methodological issue we are addressing apply to a broad swath of the research questions and hypotheses typically addressed in our field.

For simplicity, we consider that all variables are such that the use of ordinary least squares linear multiple regression (i.e., *multivariable regression* ^27^) is appropriate. Extensions to other data forms are available. ^28^ Suppose that investigators wish to test whether *X* is associated with (i.e., predicts) *Y* after controlling for *Z.* However, the investigators do not have measures of *Z.* Rather, they have measures of *Z*′, the error-contaminated or imperfect measurement of *Z.* That is why in Figure 1, *Z*′ is depicted in a rectangle whereas the other variables, which are assumed to be measured without error for this stylized example, are depicted in ellipses.

The question then becomes, will the type I error rate (i.e., sometimes called the false-positive rate) be maintained at its appropriate nominal levels if we control for *Z*′ rather than *Z* and how will any degree of bias in the significance testing be related to the reliability of *Z*′? From Figure 1, the reliability ^29^ of *Z*′ is $\alpha^{2}$ . If controlling for *Z*′ leads to unbiased inference using standard frequentist significance testing at the 0.05 significance level, then (because in this hypothetical, *X* has no effect on *Y*) the null hypothesis for the test or the association of *X* with *Y* after controlling for *Z*′ would yield significant results only 5% of the time. The (false) power (i.e., type-1 error rate inflation above the nominal significance level) of the test of the association of *X* with *Y* after controlling for *Z*′ would be the degree of bias in the significance testing procedure under these circumstances. To calculate this degree of bias or false power, we can begin by constructing the correlation matrix depicted in Table 1.

**Table 1.** Correlation matrix derived from Model 1 and Figure 1.

	*Z*′	*X*	*Y*
$Z^{\prime }$	1		
X	$\rho_{X,Z^{\prime }}=\gamma \alpha$	1	
Y	$\rho_{Z^{\prime },Y}=\alpha \beta$	$\rho_{X,Y}=\gamma \beta$	1

The correlation coefficients in the cells of the matrix in Table 1 are derived by simple application of the standard rules of path analysis. ^30^ From there, we can calculate power by writing out the *F*-test ^31^ of the association of *X* with *Y* after controlling for *Z*′. In our specific example, these yields (with N the sample size):

$$F=\left[\frac{R_{Y.X,Z^{\prime }}^{2}-R_{Y.Z^{\prime }}^{2}}{1-R_{Y.Z^{\prime }}^{2}}\right]\left[\frac{N-2-1}{2-1}\right]\;$$ (1)

For any fixed sample size and specific model, the number of covariates and sample size are constant, meaning that the rightmost term of the equation becomes irrelevant if one is only concerned with the power as a function of the measurement reliability, which is our context here. Therefore, recognizing that the non-centrality parameter and the power (false power or bias) will be monotonic functions of only the left term on the right side of equation 1, $\left[\frac{R_{Y.X,Z^{\prime }}^{2}-R_{Y.Z^{\prime }}^{2}}{1-R_{Y.Z^{\prime }}^{2}}\right]$ , we can deal only with that. This term, $\left[\frac{R_{Y.X,Z^{\prime }}^{2}-R_{Y.Z^{\prime }}^{2}}{1-R_{Y.Z^{\prime }}^{2}}\right]$ , is in fact none other than the squared partial correlation coefficient (in nonmathematical terms, a squared partial correlation coefficient can be defined as the proportion of the *remaining variance* in the dependent variable after controlling for the covariates that can be “explained” by the independent variable of interest ^32^) of *X* and *Y* after controlling for *Z*′. It is important to note that this is the squared *partial* correlation coefficient and not the squared *semi-partial* correlation coefficient [in nonmathematical terms, a squared semi-partial correlation coefficient can be defined as the proportion of the *total variance* in the dependent variable that can be “explained” by the independent variable of interest, after controlling for the covariates ^32^], because the difference in the denominators of the two coefficients can be helpful in understanding the phenomena of interest in this paper. The quantities $R_{Y.X,Z^{\prime }}^{2}\;$ and $R_{Y.Z^{\prime }}^{2}$ can then be expressed as functions of the elements of Table 1, as follows:

$$R_{y.x,z\prime }^{2}=\frac{\rho_{\mathrm{xy}}^{2}+\rho_{z\prime y}^{2}-2\rho_{\mathrm{xy}}\rho_{z\prime y}\rho_{\mathrm{xz}\prime }}{1-\rho_{\mathrm{XZ}\prime }^{2}}$$ (2)

$$R_{Y.Z^{\prime }}^{2}=\rho_{YZ^{\prime }}^{2}$$ (3)

The squared partial correlation of *Y* and *X* controlling for (i.e., after partialing out) *Z*′ is:

$$R_{Y,X.Z^{\prime }}^{2}=\left[\frac{R_{Y.X,Z^{\prime }}^{2}-R_{Y.Z^{\prime }}^{2}}{1-R_{Y.Z^{\prime }}^{2}}\right]$$ (4)

Substituting the right sides of equations 2 and 3 into the right side of equation 4 yields:

$$R_{Y,X.Z^{\prime }}^{2}=\left[\frac{\frac{\rho_{\mathrm{xy}}^{2}+\rho_{z\prime y}^{2}-2\rho_{\mathrm{xy}}\rho_{z\prime y}\rho_{\mathrm{xz}\prime }}{1-\rho_{\mathrm{XZ}\prime }^{2}}-\rho_{YZ^{\prime }}^{2}}{1-\rho_{YZ^{\prime }}^{2}}\right]$$ (5)

Equation 5 can then be re-expressed by substituting the zero-order correlations expressed as ‘ *ρ*’s in terms of the path coefficients from Figure 1 as follows:

$$R_{Y,X.Z^{\prime }}^{2}=\left[\frac{\frac{{\left(\gamma \beta \right)}^{2}+{\left(\alpha \beta \right)}^{2}-2\text{γβαβγα}}{1-{\left(\gamma \alpha \right)}^{2}}-{\left(\alpha \beta \right)}^{2}}{1-{\left(\alpha \beta \right)}^{2}}\right]$$ (6)

One can then calculate the squared partial correlation coefficient (the quantity in equation 6) as a function of the reliability of *Z*′. The reliability of *Z*′ is $\alpha^{2}$ . The first derivative of the squared partial correlation coefficient with respect to α (the square root of the reliability coefficient) is

$$\frac{dR^{2}}{\mathrm{d\alpha}}=\frac{2\alpha \left(-1+\alpha^{2}\right)\beta^{2}\gamma^{2}2-\left(1+\alpha^{2}\right)\gamma^{2}+\beta^{2}\left(-1+\alpha^{2}\left(-1+2\gamma^{2}\right)\right)}{{\left(-1+\alpha^{2}\beta^{2}\right)}^{2}{\left(-1+\alpha^{2}\gamma^{2}\right)}^{2}}$$

**Figure 2.**
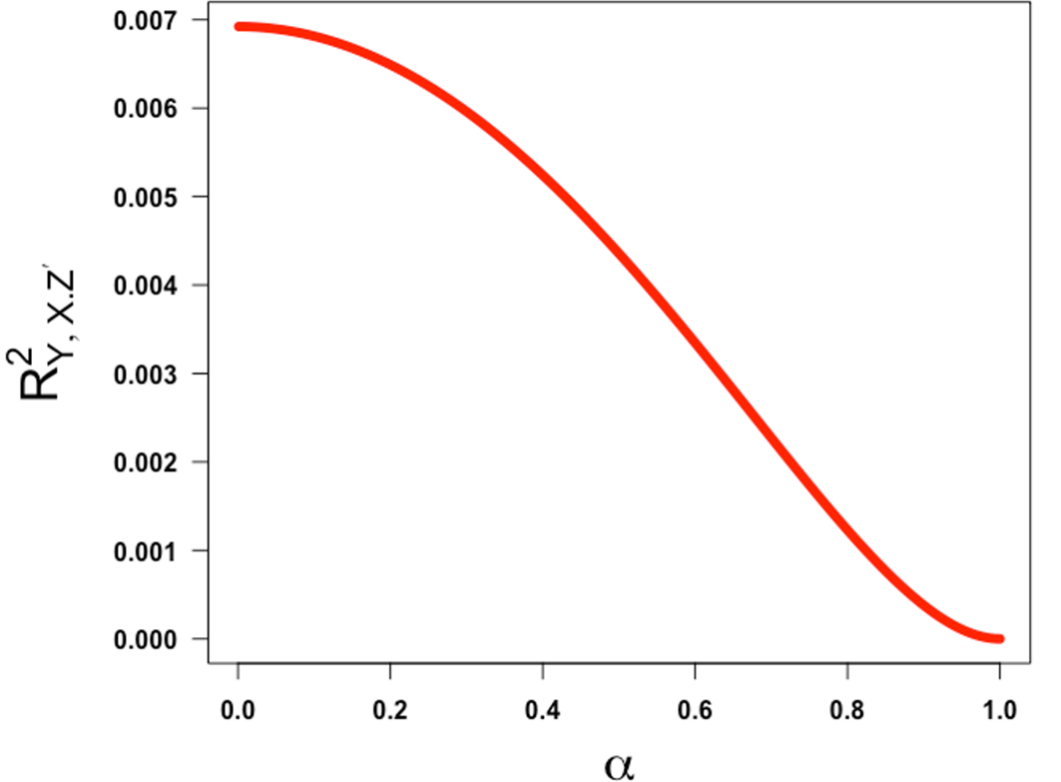
$\text{Plot of}\;R_{Y,X.Z^{\prime }}^{2}$ as a Function of *α* over the interval [0,1] assuming γ=0.64 , β=0.13 .

The first derivative of $R^{2}$ with respect to α has no real roots within the open intervals α,β,γ∈(−1,1) . The value of the derivative $\frac{dR^{2}}{d\alpha }$ for any value of α,β and, γ in (-1,1) is negative indicating that $R^{2}$ is a decreasing function of α for fixed values of β,γ in (-1,1) (see Figure 2).

All symbolic calculations for the derivative and root solutions were performed in the computer algebra system, Wolfram Mathematica 12.0.

This shows that the squared partial correlation coefficient quantifying the association between *X* and *Y* conditional on *Z*′ is always decreasing in α over the half-closed interval [0,1). This means that the lower the value of α (i.e., the lower the reliability of the confounder measurement), the higher the (spurious) partial correlation of *X* and *Y* controlling for *Z*′ will be. This illustrates the classic concept of residual confounding. This is intuitively sensible and reassuring and refutes any implication that bias due to imperfect reliability of a confounding variable increases as the reliability increases before coming back down.

**Figure 3.**
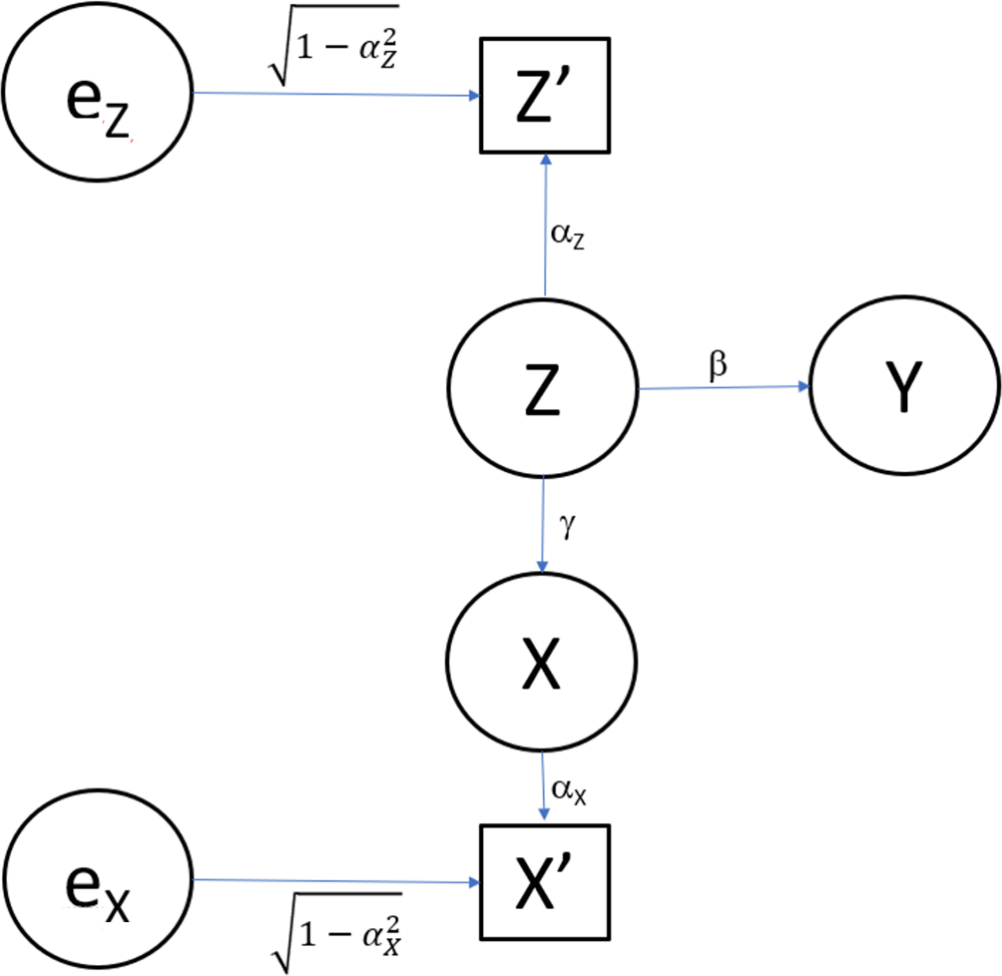
Path diagram for Model 2. The same model as in Figure 1, expect that now the exposure ( *X*) is also measured with error.

In contrast, consider the just slightly more complex model as

$$\begin{array}{c} Y=\mathrm{\beta Z}+\epsilon_{y}\\ X=\mathrm{\gamma Z}+\epsilon_{x}\\ \begin{array}{c} X^{\prime }=\alpha_{x}X+e_{x}\\ Z^{\prime }=\alpha_{z}Z+e_{z}\\ Z∼N\text{ormal}\left(0,1\right) \end{array} \end{array}$$ Model 2

also depicted in Figure 3. Again, all the error terms are assumed independent of each other and independent of *Z*. Also, we assume that all the error terms have a normal distribution with mean zero and finite variance. Without loss of generality, the error variances for *Y*, *X*, $X^{\prime },\;\text{and}\;Z^{\prime }$ are chosen so that they each have standard deviation 1. We have omitted the error terms for *Y*, *X*, and $X^{\prime }$ in Figure 3. We assume that in Model 2, only *Y*, $X^{\prime },\;\text{and}\;Z^{\prime }$ are observed, but *X* and *Z* are not observed.

The model in Figure 3 is the same as that in Figure 1 except that now *X* is also measured with error, so we observe *X*′ rather than *X.* This then produces the 3×3 correlation matrix in Table 2.

**Table 2.** Correlation Matrix Derived From Model 2 and Figure 3.

	*Z*′	*X*′	*Y*
*Z*′	1		
*X*′	$\rho_{X^{\prime },Z^{\prime }}=\alpha_{X}\gamma \alpha_{Z}$	1	
*Y*	$\rho_{Z^{\prime },Y}=\beta \alpha_{Z}$	$\rho_{X^{\prime },Y}=\beta \gamma \alpha_{X}$	1

The analogues for equations 5 and 6 from Model 1 are now equations 7 and 8 for Model 2, which are:

$$R_{Y,X^{\prime }.Z^{\prime }}^{2}=\left[\frac{\frac{\rho_{X^{\prime }Y}^{2}+\rho_{Z^{\prime }Y}^{2}-2\rho_{X^{\prime }Y}\rho_{Z^{\prime }Y}\rho_{X^{\prime }Z^{\prime }}}{1-\rho_{X^{\prime }Z^{\prime }}^{2}}-\rho_{YZ^{\prime }}^{2}}{1-\rho_{YZ^{\prime }}^{2}}\right]$$ (7)

Equation 7 can then be re-expressed substituting the zero-order correlations expressed as ‘ρ’s in terms of the path coefficients from Figure 3 as follows:

$$R_{Y,X^{\prime }.Z^{\prime }}^{2}=\left[\frac{\frac{{\left(\alpha_{X}\gamma \beta \right)}^{2}+{\left(\alpha_{Z}\beta \right)}^{2}-2\alpha_{X}\gamma \beta \alpha_{Z}\beta \alpha_{X}{\gamma \alpha }_{Z}}{1-{\left(\alpha_{X}{\gamma \alpha }_{Z}\right)}^{2}}-{\left(\alpha_{Z}\beta \right)}^{2}}{1-{\left(\alpha_{Z}\beta \right)}^{2}}\right]$$ (8)

For pedagogical purposes, let us follow Westfall and Yarkoni’s approach and let: $\alpha_{X}=\alpha_{Z}=\alpha$ .

Then, equation 8 simplifies to:

$$R_{Y,X^{\prime }.Z^{\prime }}^{2}=\left[\frac{\frac{{\left(\alpha \gamma \beta \right)}^{2}+{\left(\alpha \beta \right)}^{2}-2\text{αγβαβ}\alpha^{2}\gamma }{1-{\left(\alpha^{2}\gamma \right)}^{2}}-{\left(\alpha \beta \right)}^{2}}{1-{\left(\alpha \beta \right)}^{2}}\right]$$ (9)

One can then calculate the squared partial correlation coefficient (the quantity in equation 9) as a function of α , or the reliability of *Z*′ and *X*′, which is $\alpha^{2}$ . Taking the first derivative of the squared partial correlation coefficient with respect to the reliability coefficient, $\alpha^{2}$ , we obtain:

$$\frac{dR^{2}}{d\alpha }=\frac{2\alpha \left(-1+\alpha^{2}\right)\beta^{2}\gamma^{2}\left(-1+3\alpha^{2}+\alpha^{6}\left(-1+2\beta^{2}\right)\gamma^{2}-\alpha^{4}\left(2\beta^{2}+\gamma^{2}\right)\right)}{{\left((-1+\alpha^{2}\beta^{2}\right)}^{2}{\left(-1+\alpha^{4}\gamma^{2}\right)}^{2})}$$

**Figure 4.**
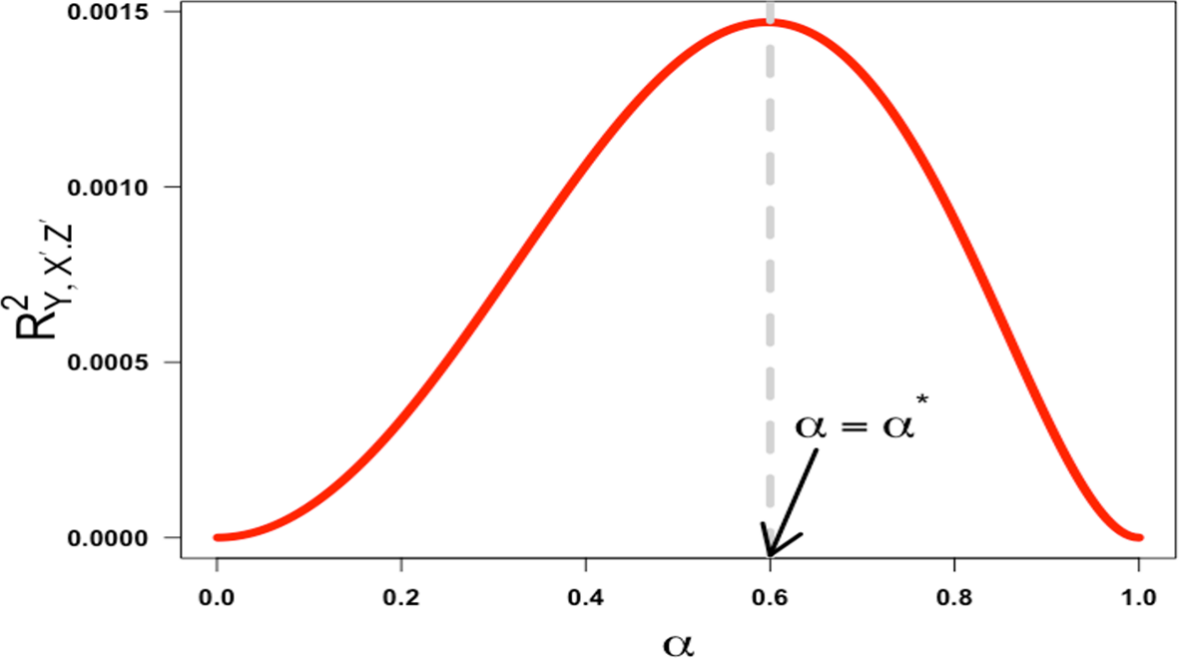
$\text{Plot of}\;R_{Y,X^{\prime }.Z^{\prime }}^{2}$ against α when γ=0.19,β=0.50 . The critical point is labeled $\alpha =\alpha^{*}$ and the regions are identified as $\alpha <\alpha^{*}$ to the left of $\alpha^{*}$ and $\alpha >\alpha^{*}$ to the right of $\alpha^{*}$ .

Setting the derivative equal to zero and solving for α under the constraints that α,β,γ are all in the interval (-1,1) results in a unique critical point, $\alpha^{*}\in \left(0,1\right)$ . Because $\frac{dR^{2}}{d\alpha }$ is an even function of α,β,γ , there is a symmetric critical point, $-\alpha^{*}\in \left(-1,0\right)$ . The region where α<0 is a reflection of the region α>0 across the y-axis; therefore, we can restrict our analysis to the region, α>0 . In this region, with α>0 , the derivative is positive for $\alpha <\alpha^{*}$ and negative for $\alpha >\alpha^{*}$ in the interval (0,1), verifying that the function increases before the critical point and decreases after. As a result, the squared partial correlation coefficient quantifying the association between *X*′ and *Y* conditional on *Z*′ is not monotonic in α over the half-closed interval [0,1). This is illustrated in Figure 4 below with (γ=0.19,β=0.50 ).

This would seem to support Westfall and Yarkoni’s statement that “the effect of reliability on error rates is even less intuitive: there is a non-monotonic relationship, such that type 1 error approaches 5% [the nominal rate they set] when reliability nears 0 or 1, but is highest when reliability is moderate”. ^6^ What is not made clear in their paper is the reliability of what? Specifically, their statement is not true when referring to a single reliability coefficient. It is only true because they have constrained the reliability of the exposure measurement and the reliability of the confounder measurement to have the same value. If we decouple them (as there is no *a priori* reason that they must have the same value), and return to equation 8 instead of equation 9, we can see that, when *Z* is a confounder, the squared partial correlation coefficient (the quantity in equation 8) as a function of $\alpha_{Z}$ , or the reliability of *Z*′, is always increasing as a function of $\alpha_{X}$ , or the reliability of *Z*′.

This leads to the insight that it is the *relative* values of $\alpha_{Z}$ and $\alpha_{X}$ that determine this pattern. To make this clear, we re-express $\alpha_{X}=\omega \alpha_{Z}$ and then substitute into equation 8 to yield:

$$R_{Y,X^{\prime }.Z^{\prime }}^{2}=\left[\frac{\frac{{\left(\omega \alpha_{z}\gamma \beta \right)}^{2}+{\left(\alpha_{Z}\beta \right)}^{2}-2\omega \alpha_{z}\gamma \beta \alpha_{Z}\beta \omega \alpha_{z}\gamma \alpha_{Z}}{1-{\left(\omega \alpha_{z}\gamma \alpha_{Z}\right)}^{2}}-{\left(\alpha_{Z}\beta \right)}^{2}}{1-{\left(\alpha_{Z}\beta \right)}^{2}}\right]$$

in reduced form we have $R_{\left(Y,X^{\prime }.Z^{\prime }\right)}^{2}=\left[\frac{{\left(\alpha_{z}\beta \omega \gamma \right)}^{2}\left(1-\alpha_{z}^{2}\left(2-\alpha_{z}^{2}\right)\right)}{\left(1-{\left(\omega \alpha_{z}^{2}\gamma \right)}^{2}\right)\left(1-{\left(\alpha_{z}\beta \right)}^{2}\right)}\right]$

provided that $\omega <1/\left(\gamma \alpha_{z}^{2}\right)$

**Figure 5.**
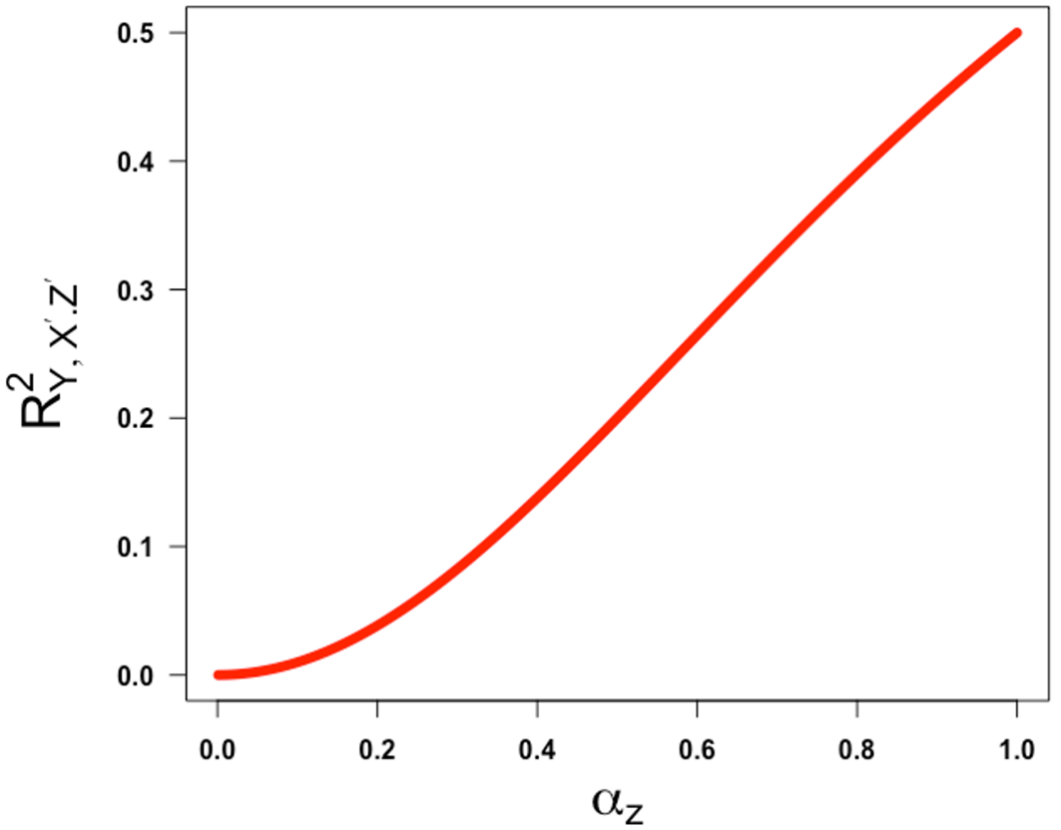
$\text{Plot of}\;R_{Y,X^{\prime }.Z^{\prime }}^{2}$ against $\alpha_{z}$ , the square root of the reliability of the confounder measurement. Partial correlation is obtained assuming γ = β=1 and ω = -1.

**Figure 6.**
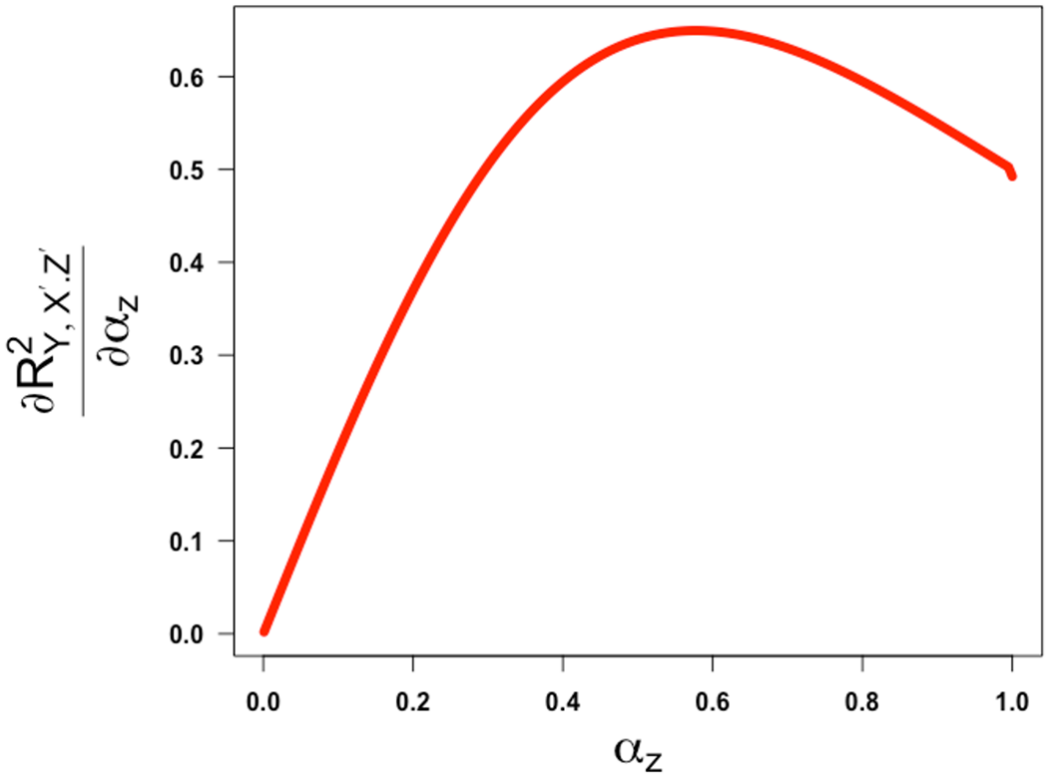
$\text{Plot of the first derivative of}\;R_{Y,X^{\prime }.Z^{\prime }}^{2}$ against $\alpha_{z},$ the square root of the reliability of the confounder measurement. The partial correlation is obtained assuming γ = β=1 and ω = -1.

Using this expression, we can make the following observations. This expression is a product of all positive terms hence is positive. If we relax the assumptions made above and only impose that $\alpha_{z}\in \left(0,1\right)$ and γ and β are both set at 1.0 and ω = -1, then we can show that the partial correlation above is monotone increasing with the reliability $\alpha_{z}$ and converges to ½ as $\alpha_{z}$ get close to 1 from the left ( $\alpha_{z}<1).$ Figures 5 and 6 show the plots of the partial correlation and its first derivative, respectively, as a function of $\alpha_{z}$ when γ and β are both set at 1.0 and ω = -1, both illustrating that the partial correlation is a non-decreasing function of $\alpha_{z}$ in these settings. Further, it turns out that if we assume $\alpha_{X}=\omega \alpha_{Z}+a_{0}$ and we set ω=0.8 and $a_{0}=0.2$ while keeping γ and β both at 1.0, the partial correlation is also monotone in $\alpha_{z}\left(\text{plot not shown}\right)$ . ^43^ We provide a small R code to reproduce the plots presented in this paper along with the case where we set ω=0.8 and $a_{0}=0.2$ while keeping γ and β both at 1.0. ^43^

What these derivations and illustrations show is that the degree of ‘false power’ or bias can actually goes up as one moves from a completely unreliable measure of a confounding variable to a modestly reliable measure of a confounding variable as Westfall and Yarkoni opined, but *only if one artificially constrains the reliability of the exposure measurement to also be increasing as* a function of the reliability of the confounder measurement. This is because, as we showed previously, ^33^ although the numerator of a ratio and the denominator of a ratio may both move toward their “proper” values monotonically as a function of a single variable, they may do so at different rates such that the ratio may change non-monotonically.

## Implications for optimal design

Many epidemiologic studies involve observational (not interventional) studies and as such are not under experimental control. ^34^ Such observational data are often prone to measurement error, missing data, and confounding. While it is well known that measurement error associated with a single exposure, X, under some very specific circumstances, leads to attenuated associations in simple linear regression, less is known about how measurement error influences estimation, testing validity, and optimal study designs when investigators are interested in modeling the association between the exposure, and an outcome, Y, after controlling for Z, a confounder, where are all measured with error and complex error structures and analysis methods may prevail.

In nutritional studies involving estimation of dietary exposures on disease outcome models, linear regression calibration is the most applied method. ^35^ Linear calibration-based methods to measurement error correction involve two-stage models where replacement values for the true dietary exposure are simulated or obtained from its conditional distribution given the measured value and association between the simulated measures of the exposures and the outcome are estimated in the second stage. ^36^ Under the linear calibration approaches, non-differential measurement error and uncorrelated errors between the dietary and reference instruments are assumed. ^35^ In models where a confounder and an exposure are both measured with error or when multiple covariates are measured with error, multivariable regression calibration is often used in nutritional studies. ^37^ The multivariable regression calibration approach is performed under assumptions of random within-person errors or under combinations of random error. In general, the effect of measurement error in a single mis-measured or imprecisely observed exposure is to attenuate its effects on health outcomes. However, how measurement error in both a confounder and exposure influence their estimated effects on the dependent variable is less clear. Measurement error can modify or mask the effects of a confounder when both the confounder and exposure are error prone. ^34^

While efforts have been made to develop statistical approaches that correct for measurement error to reduce biases in the estimation of exposure effects, less has been done to determine how the presence of measurement error in an exposure or in both exposure and confounder influences optimal study designs. By optimal study design, we mean the design which minimizes some loss function (e.g., some weighted function of quantities of interest such as power, type 1 error rate, bias, financial cost, study duration, etc.) subject to user-defined constraints. ^38^ Spiegelman and Gray ^39^ developed cost-efficient and statistically powerful cohort study designs for continuous exposures measured with error in binary logistic regression models. The authors proposed three ways to optimize study designs in the presence of errors. These include the inclusion of an internal validation study, external validation of subsamples obtained from other studies, and the use of better exposure methods for assessment. ^39^

Such thinking invites several questions for future research when we wish to test for the association between an independent variable and a dependent variable after controlling for a third variable, and all may be measured with error.

• A first question is a meta-research question ^40^: What is the state of practice? When investigators apply measurement error correction procedures, do they typically do so for *X*, *Z*, *Y*, or some combination? Although systematic reviews of related questions have appeared, ^35^ we are not aware of one that has answered this exact question.
• A second question is how does applying such measurement error corrections affect power and bias if applied to various combinations of *X* vs *Y* vs *Z*?
• Third, do the answers to the second question suggest under which circumstances, to what extent, and for which variables ( *X*, *Y*, or *Z*) investigators should invest their resources in improving the reliability of if the possibility of increasing reliability of each at the cost of finite funds exists? Such inquires might lead to the counterintuitive finding that under some circumstances one should invest more in studying the nuisance variables one is not interested in than the putative causal factors in which one is interested.

## General implications

Without knowing the exact nature of any phenomenon or set of phenomena we are studying, we cannot know exactly which situation will prevail. If we did, we would not need to undertake the research. However, we can say from general knowledge of the measurement properties of variables frequently used in nutrition and epidemiologic research, and of the magnitude of associations typically studied in epidemiology and nutrition and obesity, that the hypothetical scenarios we have evaluated above are within the realm of the typical association study. Therefore, it seems plausible that not only is measurement error a substantial concern in our models but so are statements that may be patently untrue, such as that an association actually becomes more significant when controlling for a plausible confounder or that measurement error only attenuates effects. We thus may need to take observational research with more than a considerable grain of salt when drawing causal inferences. Yet further, the plausibility of our hypothetical example suggests the importance of not merely acknowledging measurement error as most of us do in our articles on nutrition epidemiology, but actually building formal measurement error corrections into the analysis as described elsewhere. ^41^ Included in overall implications for research in nutritional epidemiology are the untrue yet-seeming implicit default assumptions that: 1) there is a linear or monotonic dose-response of risk with intake of a food or nutrient; 2) confounders act independently of one another, additively, linearly, with predictable effect, when they clearly do not, accentuating concerns about the ignoring of potential interactions of multiple confounders that are either not measured at all or not measured accurately ^42^; and 3) unmeasured or poorly measured confounders can be dismissed as serious threats to statistical conclusion validity because it is implausible that a confounder or set of confounders could imbue sufficient bias to produce the observed association, when in fact large samples produce large power to mistakenly detect small biases as actual associations or effects. Finally, our illustrations and derivations point out the importance of evaluating the quality of our procedures and improving them when possible.

As outlined in the beginning, the topic of measurement error in covariates has critical application for nutritional epidemiology around the practice of determining nutrient and energy intake and using it for developing nutritional guidance and policies. It is unclear what role interpretations based on inaccurate measurements have played in national and local health-related policies or health care guidance for individuals. It should be recognized that a great need exists for accurately determining nutrient or energy intake. This need will not become a priority if we continue to accept inaccurate data as “good enough” for the purpose. Therefore, moving forward, we need to discontinue the use of methods that generate decidedly inaccurate data. Next, we urge that concerted efforts be made in redirecting resources to developing methods that objectively measure nutrient and energy intake with high fidelity and for long periods of time. Until such time, we need to be prepared to accept the counterintuitive conclusion that under some circumstances, in our efforts to improve the reliability or power of our methods, *sometimes nothing is better than something.*

## Ethics and consent

Ethical approval and consent were not required.

## Data Availability

No data are associated with this article. Zenodo: rszoh/ReliabME: Version 1. https://zenodo.org/doi/10.5281/zenodo.12639728. ^43^ Analysis code available from: https://github.com/rszoh/ReliabME/. Data are available under the terms of the Creative Commons Attribution 4.0 International license (CC-BY 4.0).
