# Supplementary material for: Adjusting for covariates representing potential confounders, mediators, or competing predictors in the presence of measurement error: Dispelling a potential misapprehension and insights for optimal study design with nutritional epidemiology examples
Source: F1000Res. 2025 May 19;13:827. Originally published 2024 Jul 24. [Version 2] doi: 10.12688/f1000research.152466.2 (PMC12181766; doi:10.12688/f1000research.152466.2)
Supplement: Peer review history

# Reviewer report: Reviewer response for version 2

**DOI:** 10.5256/f1000research.181984.r388902

**Published:** 2025-07-21

**Authors:** Didier Brassard

**Affiliations:** 1 McGill University, Montreal, Québec, Canada

**Summary**

 Authors used simulation data to examine a potential “contamination” effect when controlling for a confounding variable measured with error, which could amplify type 1 error rate for exposure-outcome association. Authors found that in a optimistic setting (one covariate measured with error), bias follows expectations. However, a more realistic setting (both exposure and covariate measured with error) showed that the degree of “false power” can actually increase given the reliability of the covariate measurement. Of note, it is the first time I review this study which has already undergone revisions. This article is relevant given the importance of measurement error in nutrition. I have a few comments to help readers better understand the setting and the implication. Thank you for the opportunity to review this work. I have not reviewed the equations beyond model description due to lack of technical expertise on the matter.

 As a general comment, the study findings highlight a problem that occurs when no measurement error correction is performed if I understood correctly. Yet, the discussion draws on these findings to support statement about the field of nutritional epidemiology in general. It seems relevant to better position the context of these findings (see first major comment) and discuss available options (see second major comment).

**Major**

 A few additional statements would help clarify the setting for readers. First, it would be relevant to clarify which type of measurement error is of interest earlier in the article (e.g., classical, linear). Second, please indicate which dietary assessment instrument is prone to these errors in nutritional epidemiology (e.g., short-term such as 24-h dietary recalls or long-term) and ideally with a qualitative quantification of the extent. Third, although the examples are clear in that regard, please ensure the number of variables measured with error is clear (eg introduction). The latter is relevant because the impact of (classical) measurement error is hard to predict in a multivariable model for example, as both over or under-estimation of effect size can occur relative to the same variables measured without error. Fourth, please indicate if errors were assumed to be non-differential in the “Derivation” section. Finally, the simulation primarily addressed power to detect a relationship and type 1 error rate. Could authors also allude to or describe the potential impact on relationship estimates (e.g., regression coefficient)?

 The current conclusion (eg, “we urge that concerted efforts be made in redirecting resources to developing methods that objectively measure nutrient and energy intake with high fidelity and for long periods of time”) seems to suggest there is no solution currently available. Yet, it has been recognized for 30y+ that improvement in dietary assessment won’t be through discovery of the ideal instrument (accurate and precise) but rather through better understanding of errors and statistical approaches to account for these errors (Beaton, 1994). Accordingly, much work has been done to better understand how different errors affect common dietary assessment instruments such as FFQ and 24-h dietary recalls, see (Thompson et al., 2015) and papers by Freedman et al. (Freedman et al., 2014; Freedman et al., 2015). A workable solution is the “coping strategy” where one uses for the least-biased instrument (i.e., 24-h dietary recalls) in combination with statistical methods to account for classical (random) errors, see (Kirkpatrick et al., 2022) for a description in the surveillance setting. My recommendations are as follows. First, based on the study results, clarify which instrument is not fit for purpose (eg, screeners, FFQ, food records or 24-h dietary recalls) and for which analytical task (detecting association? estimating effect size? visual assessment?). Second, expand the discussion to highlight which solution is available, i.e., the “coping strategy” of using the least-biased instrument in combination with measurement error correction for random errors. Third, clarify which setting or design would yield inaccurate findings, and which setting would yield findings that are plausibly more accurate. More precisely, clarify which instrument or combination of instruments, (recovery) biomarker and measurement error correction, and repeated data collection is good enough or rather insufficient.

**Minor**

 Introduction. “For example, a study pooled data from five large studies that had examined the validity of food-frequency questionnaires and 24-h dietary recalls against recovery biomarkers *… *”. I recognize that authors are referring to these publications: Freedman et al. Am J Epidemiol 2015. PMID: 25787264; Freedman et al. Am J Epidemiol 2014; PMID. 24918187. Yet, I can’t find the citation in the references. Please confirm or revise citations.

 Introduction: “that they could be adjusted by a common factor.” Is this statement referring to energy adjustment, for example calculating protein density (% energy)? I think it should be mentioned more explicitly if that is the case or clarified. Furthermore, if this is indeed referring to energy adjustment, work by Freedman et al. did find that energy adjustment slightly improved correlation with true intake for FFQ, although the FFQ measurement was still far from truth. I suggest authors revise this paragraph of the introduction to add more nuances to their argument.

 The nutrition example in section ***Confounding, direct effects in mediation, and incremental predictive validity *** is useful to conceptualize the problem. However, the example raises further complexity. For example, the example of model 1 includes energy (X), blood pressure (Y) and sodium (Z). Typically, both energy intake and sodium would be measured with correlated error if they were obtained using the same self-report dietary assessment instrument. I suggest authors expand their example by describing how the energy intake would be measured or if the assumption of no error is simply to provide a first simple example to illustrate the concept. Then, in example 2, I suggest indicating which instrument would be used when X is measured with (classical?) error. For example, using recovery biomarkers, i.e., doubly labelled water?

 Regarding the conclusion for example 2 (“ *but only if one artificially constrains the reliability of the exposure measurement to also be increasing as a function of the reliability of the confounder measurement*.”), could authors provide a complementary explanation using a concrete example? In other words, what assumptions for dietary assessment instruments would have to be true for that observation to happen?

 “we need to discontinue the use of methods that generate decidedly inaccurate data.” Please state more explicitly which instrument has been shown to provide inaccurate data, for which purpose and provide reference to support this statement or draw on study results.

**“**We thus may need to take observational research with more than a considerable grain of salt when drawing causal inferences” This statement is not entirely consistent with the sentence before. For instance, if statements about the potential effect of measurement error are inaccurate in scientific articles, then, the solution is to avoid making incorrect statements about the direction of measurement error to avoid misleading readers; not necessarily to avoid making causal inferences altogether.

Is the work clearly and accurately presented and does it cite the current literature?

Partly

If applicable, is the statistical analysis and its interpretation appropriate?

I cannot comment. A qualified statistician is required.

Are all the source data underlying the results available to ensure full reproducibility?

Yes

Is the study design appropriate and is the work technically sound?

Yes

Are the conclusions drawn adequately supported by the results?

Partly

Are sufficient details of methods and analysis provided to allow replication by others?

Yes

Reviewer Expertise:

Nutritional epidemiology, nutritional surveillance, (classical) measurement error correction methods in nutrition

I confirm that I have read this submission and believe that I have an appropriate level of expertise to confirm that it is of an acceptable scientific standard, however I have significant reservations, as outlined above.

## References

[1] : Approaches to analysis of dietary data: relationship between planned analyses and choice of methodology. *The American Journal of Clinical Nutrition* .1994;59(1) : 10.1093/ajcn/59.1.253S 253S-261S 10.1093/ajcn/59.1.253S 8279436

[2] : Pooled Results From 5 Validation Studies of Dietary Self-Report Instruments Using Recovery Biomarkers for Energy and Protein Intake. *American Journal of Epidemiology* .2014;180(2) : 10.1093/aje/kwu116 172-188 10.1093/aje/kwu116 PMC408234124918187

[3] : Using Short-Term Dietary Intake Data to Address Research Questions Related to Usual Dietary Intake among Populations and Subpopulations: Assumptions, Statistical Techniques, and Considerations. *Journal of the Academy of Nutrition and Dietetics* .2022;122(7) : 10.1016/j.jand.2022.03.010 1246-1262 10.1016/j.jand.2022.03.010 35283362

[4] : The National Cancer Institute’s Dietary Assessment Primer: A Resource for Diet Research. *Journal of the Academy of Nutrition and Dietetics* .2015;115(12) : 10.1016/j.jand.2015.08.016 1986-1995 10.1016/j.jand.2015.08.016 PMC466311326422452

[5] : Pooled Results From 5 Validation Studies of Dietary Self-Report Instruments Using Recovery Biomarkers for Potassium and Sodium Intake. *American Journal of Epidemiology* .2015;181(7) : 10.1093/aje/kwu325 473-487 10.1093/aje/kwu325 PMC437176625787264

# Reviewer report: Reviewer response for version 2

**DOI:** 10.5256/f1000research.181984.r385859

**Published:** 2025-06-20

**Authors:** Nicola Bondonno

**Affiliations:** 1 The Danish Cancer Society Research Centre, Copenhagen, Denmark

Approved with no further comments

Is the work clearly and accurately presented and does it cite the current literature?

Partly

If applicable, is the statistical analysis and its interpretation appropriate?

I cannot comment. A qualified statistician is required.

Are all the source data underlying the results available to ensure full reproducibility?

Yes

Is the study design appropriate and is the work technically sound?

Yes

Are the conclusions drawn adequately supported by the results?

Yes

Are sufficient details of methods and analysis provided to allow replication by others?

Yes

Reviewer Expertise:

Nutritional epidemiology

I confirm that I have read this submission and believe that I have an appropriate level of expertise to confirm that it is of an acceptable scientific standard.

# Reviewer report: Reviewer response for version 1

**DOI:** 10.5256/f1000research.167231.r330829

**Published:** 2024-11-26

**Authors:** Tolulope Sajobi

**Affiliations:** 1 University of Calgary, Calgary, Canada

Thank you for the opportunity to review the manuscript by Zoh and colleagues on the implications of adjusting for covariates measured with error on strength of association between an exposure and covariates. The authors, through simple but elegant mathematical derivations  demonstrated the implications of measurement error on Type I error and bias in the association between an exposure (e..g, nutrient) and outcome.  Specifically, the authors examined the impact of power and bias under two scenarios

1. When the confounding variable Z is measured with error but not the exposure variable X.
2. When the confounding variable Z and exposure variable X are both measured with errors.

The authors showed that there is a non-monotonic relationship between the degree of measurement error and the Type I error and bias in the estimation of the association between an exposure and outcome. The authors also outlined some ideas for future research.

 As a minor revision, the authors should comment on the  possible conclusion from extending this investigation to generalized linear models, especially with a non-linear link function.

Is the work clearly and accurately presented and does it cite the current literature?

Yes

If applicable, is the statistical analysis and its interpretation appropriate?

Yes

Are all the source data underlying the results available to ensure full reproducibility?

Yes

Is the study design appropriate and is the work technically sound?

Yes

Are the conclusions drawn adequately supported by the results?

Yes

Are sufficient details of methods and analysis provided to allow replication by others?

Yes

Reviewer Expertise:

Biostatistics; epidemiology;

I confirm that I have read this submission and believe that I have an appropriate level of expertise to confirm that it is of an acceptable scientific standard.

# Response

**Published:** 2025-05-10

**Authors:** Roger Zoh

**Affiliations:** Epidemiology and Biostatistics, Indiana University Bloomington, Bloomington, Indiana, USA

Thank you for the opportunity to review the manuscript by Zoh and colleagues on the implications of adjusting for covariates measured with error on strength of association between an exposure and covariates. The authors, through simple but elegant mathematical derivations demonstrated the implications of measurement error on Type I error and bias in the association between an exposure (e.g., nutrient) and outcome. 

 Specifically, the authors examined the impact of power and bias under two scenarios

  

1. When the confounding variable Z is measured with error but not the exposure variable X.
2. When the confounding variable Z and exposure variable X are both measured with errors.

The authors showed that there is a non-monotonic relationship between the degree of measurement error and the Type I error and bias in the estimation of the association between an exposure and outcome. The authors also outlined some ideas for future research.

 As a minor revision, the authors should comment on the possible conclusion from extending this investigation to generalized linear models, especially with a non-linear link function.

Response:

 The results discussed here are not readily extendable to the case of generalized linear models with non-linear link functions. We added the following text to the paper:

 The results obtained here were developed in the context of linear models and   are not readily extendable to the case of generalized linear models with non-linear link functions. (Huang, 2016; Muller et al., 1992; Neyestanak, 2021; Rocci, 2002; Schafer, 1987)."

# Reviewer report: Reviewer response for version 1

**DOI:** 10.5256/f1000research.167231.r336327

**Published:** 2024-11-11

**Authors:** Nicola Bondonno

**Affiliations:** 1 The Danish Cancer Society Research Centre, Copenhagen, Denmark

The final sentence of the introduction suggests that the target audience for this paper includes researchers, reviewers, and editors in nutrition and obesity research. However, the level of statistical detail makes the content accessible only to those with an advanced statistical background. As a nutritional epidemiologist with a strong grasp of statistics, I struggled to follow the derivations from the first sentence onward. Given this, I will not comment on the technical aspects of the math. However, as someone within your target audience, I found that the paper lacked clear takeaways, which could be improved for better accessibility and impact. I'll provide a few suggestions that may help enhance clarity.

 The figures are informative, yet the material could be made even more approachable with a consistent, specific example that guides both experts and non-experts throughout the paper. For instance, the approach taken in Tomova et al. (2022) may serve as a useful model, where a single example runs consistently across sections (refer 1). Currently, you use a few different examples, but consolidating to one example could help maintain continuity and comprehension.

 If I understand correctly, the association shown in Figures 4, 5, and 6 (as proposed by Westfall and Yarkoni) only holds when the reliability of the exposure increases as the reliability of the confounder increases. This isn’t typically expected in real-world settings and clarifying this point with an example would enhance understanding. In the introduction, you state, “Here we provide an explanation of the phenomenon in simple scenarios so that researchers, reviewers, and editors in the nutrition and obesity research communities can see that the conclusion about non-monotonicity of bias as a function of covariate reliability holds only under specific and arguably contrived circumstances.” While this is a valuable aim, I didn’t find this point sufficiently addressed in the Discussion. Your conclusions focus primarily on the need to improve the accuracy of exposure and confounder measurements, which is important but misses practical advice for researchers working with pre-collected data, where measurement error is often unknown. Can you clarify for readers in this situation—what should they do or acknowledge in their studies? Expanding on this in the Discussion would make your conclusions more actionable.

 I also disagree with the statement: “Included in overall implications for research in nutritional epidemiology are the untrue yet-seeming implicit default assumptions that: 1) there is a linear or monotonic dose-response of risk with intake of a food or nutrient.” Most quality papers in the field do not assume this and instead use splines or categorize exposures to avoid such assumptions.

Is the work clearly and accurately presented and does it cite the current literature?

Partly

If applicable, is the statistical analysis and its interpretation appropriate?

I cannot comment. A qualified statistician is required.

Are all the source data underlying the results available to ensure full reproducibility?

Yes

Is the study design appropriate and is the work technically sound?

Yes

Are the conclusions drawn adequately supported by the results?

Yes

Are sufficient details of methods and analysis provided to allow replication by others?

Yes

Reviewer Expertise:

Nutritional epidemiology

I confirm that I have read this submission and believe that I have an appropriate level of expertise to confirm that it is of an acceptable scientific standard, however I have significant reservations, as outlined above.

## References

[1] : Adjustment for energy intake in nutritional research: a causal inference perspective. *Am J Clin Nutr* .2022;115(1) : 10.1093/ajcn/nqab266 189-198 10.1093/ajcn/nqab266 34313676 PMC8755101
